# Continuing Professional Development—Radiation Therapy

**DOI:** 10.1002/jmrs.879

**Published:** 2025-03-19

Maximise your continuing professional development (CPD) by reading the following selected article and answering the five questions. Please remember to self‐claim your CPD and retain your supporting evidence. Answers will be available via the QR code and published in JMRS—Volume 72, Issue 4, December 2025.

## Artificial Intelligence in Radiation Therapy Treatment Planning: A Discrete Choice Experiment

M. Lewandowska, D. Street, J. Yim, S. Jones, R. Viney, *Journal of Medical Radiation Sciences* (2025), https://doi.org/10.1002/jmrs.843.

1. What is the primary focus of the study?
  - The economic impact of cancer treatment
  - Preferences for adopting artificial intelligence (AI) in radiation therapy treatment planning
  - The effectiveness of manual radiation therapy techniques
  - Patient outcomes in radiation oncology
2. What method did the researchers use to collect their data?
  - Qualitative interviews
  - Focus group discussions
  - Discrete choice experiment
  - Randomised controlled trials
3. Based on the study, which feature of AI systems did radiation oncology professionals prefer the most?
  - Reduced accuracy
  - Systems that require more time per patient
  - Systems that provide the greatest time savings and explain the AI reasoning
  - Fully autonomous systems
4. What does the study indicate about radiation oncology professionals' preferences regarding AI automation?
  - A preference for fully autonomous AI systems
  - A preference for assistive AI systems requiring human verification
  - No preference for automation
  - A preference for manual systems only
5. How many respondents completed the online discrete choice experiment in the study?
  - 50
  - 82
  - 112
  - 137

## Answers

Scan this QR code to find the answers.

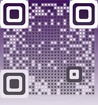

## References

[1] H. S. J. Chew , and P. Achananuparp , “Perceptions and Needs of Artificial Intelligence in Health Care to Increase Adoption: Scoping Review,” Journal of Medical Internet Research 24, no. 1 (2022): e32939, 10.2196/32939. 35029538 PMC8800095

[2] M. Kawamura , T. Kamomae , M. Yanagawa , et al., “Revolutionizing Radiation Therapy: The Role of AI in Clinical Practice,” Journal of Radiation Research 65, no. 1 (2024): 1–9, 10.1093/jrr/rrad090. 37996085 PMC10803173

[3] G. R. Sarria , F. Kugel , F. Roehner , et al., “AI‐Based Auto‐Segmentation: Advantages in Delineation, Absorbed Dose‐Distribution and Logistics,” Advances in Radiation Oncology 9, no. 3 (2023): 101394, 10.1016/j.adro.2023.101394. 38292888 PMC10823084

